# Hedgehog Signaling in Lung Cancer: From Oncogenesis to Cancer Treatment Resistance

**DOI:** 10.3390/ijms19092835

**Published:** 2018-09-19

**Authors:** Etienne Giroux-Leprieur, Adrien Costantini, Vivianne W. Ding, Biao He

**Affiliations:** 1 Department of Respiratory Diseases and Thoracic Oncology, APHP-Hopital Ambroise Pare, 92100 Boulogne-Billancourt, France; Etienne.giroux-leprieur@aphp.fr (E.G.-L.); Adrien.Costantini@aphp.fr (A.C.); 2 EA 4340, UVSQ, Université Paris-Saclay, 92100 Boulogne-Billancourt, France; 3 Thoracic Oncology Program, Department of Surgery, Helen Diller Family Comprehensive Cancer Center, University of California, San Francisco, CA 94143, USA; vivianne.ding@ucsf.edu

**Keywords:** Sonic Hedgehog, lung cancer, oncogenesis, cancer stem cell, chemoresistance, Epidermal Growth Factor Receptor (EGFR), tyrosine kinase inhibitor, radiotherapy, immune checkpoint inhibitor

## Abstract

Hedgehog signaling pathway is physiologically activated during embryogenesis, especially in lung development. It is also reactivated in many solid tumors. In lung cancer, Hedgehog pathway is closely associated with cancer stem cells (CSCs). Recent works have shown that CSCs produced a full-length Sonic Hedgehog (Shh) protein, with paracrine activity and induction of tumor development. Hedgehog pathway is also involved in tumor drug resistance in lung cancer, as cytotoxic chemotherapy, radiotherapy, and targeted therapies. This review proposes to describe the activation mechanisms of Hedgehog pathway in lung cancer, the clinical implications for overcoming drug resistance, and the perspectives for further research.

## 1. Introduction

Hedgehog pathway has been highly conserved during development of species [1,2]. It is a major pathway in mammalians, involved notably during embryogenesis [2]. Three different proteins have been described in Hedgehog (Hh) pathway: Sonic, Indian, and Desert Hedgehog, differentially expressed according to the species. In humans, Sonic Hedgehog (Shh) is the main protein. Shh pathway is activated at the early steps of embryogenesis, during organogenesis [3,4,5]. It is one of the key regulatory pathways during limb formation, determining fingers’ number and identity, and during development of the central nervous system, inducing the dorsoventral axis on the neural crest [2]. In the lung, Shh pathway is involved in bronchial budding. Shh is secreted by epithelial cells, with a paracrine effect on mesenchymal cells, acting as a spatial regulator of bronchial bud formation. Mice models of Shh pathway inhibition (Shh^−/−^, Ptch1^−/−^, Gli2^−/−^, and Gli3^−/−^ knock-out) induce severe lung malformations, with hypoplasia and tracheal malformations and non-viable phenotypes [3,6,7,8,9]. Several lung diseases were found to be related to Shh activation. Lung fibrosis is characterized by epithelial lesions, with induction of epithelial-mesenchymal transition (EMT) [10]. The proliferation of fibroblastic cells leads to collagen deposit and destruction of the alveolar organization [11]. Several studies have raised the major role of Shh pathway in lung fibrosis. Shh, Smoothened (Smo), Patched (Ptch), Gli1 are overexpressed in lung tissues in idiopathic lung fibrosis (ILF) [12,13,14,15]. As in lung embryogenesis, Shh seems to be secreted by epithelial cells, with autocrine and paracrine activities on mesenchymal cells. In mice models, Shh activates mainly fibroblasts and myofibroblasts after bleomycin or fluorescein isothiocyanate (FITC) induction of lung fibrosis [13,16,17]. Pirfenidone, a drug given in ILF, induces anti-fibrotic effects through Shh inhibition in patients with lung fibrosis [18,19]. There is therefore a close relation between Shh pathway and lung, in physiological conditions but also in lung diseases. This review aims to describe the activation of Shh pathway in lung cancer, and its implication in oncogenesis, through cancer stem cells (CSCs), and in resistance to anti-tumor treatments.

## 2. Canonical and Non-Canonical Shh Pathway Activation

**Figure 1:**
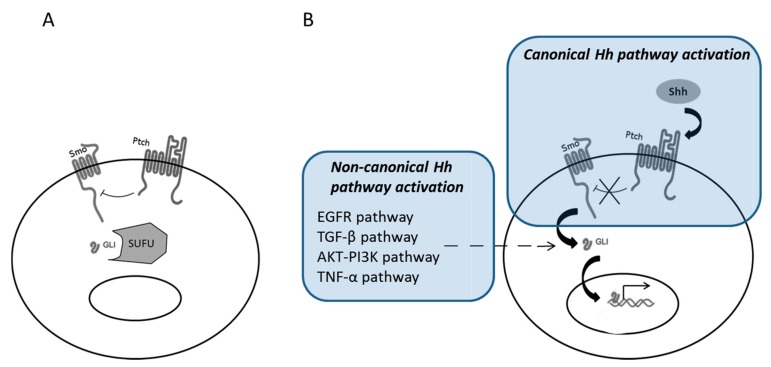
Hedgehog (Hh) pathway activation. At basal state, Ptch inhibits Smo, and Gli proteins are kept in the cytosol par suppressor of fused homolog (SUFU) (**A**). In canonical Hh pathway activation, Sonic Hedgehog (Shh) binds to Ptch, Smo is activated, leading to translocation of Gli in the nucleus and transcription of target genes. Non-canonical Hh Pathway activation involves Epidermal Growth Factor receptor (EGFR), TGF-β, AKT-PI3K, and TNF-α pathways, with direct activation of Gli without Smo activation (**B**).

The activation of Shh pathway can be induced through a canonical and a non-canonical mode (Figure 1). The canonical activation is based on the binding of Shh on its receptor, Ptch. Shh is a 45 kDa-protein, physiologically cleaved in the cytosol into two peptides (N-terminal and C-terminal peptides) [20]. N-terminal peptide is then modified by the addition of lipid (palmitoyl and cholesterol) molecules [21,22]. The N-terminal peptide is thereafter anchored in the cell membrane, before its secretion to the extracellular space, leading to paracrine activity [23,24]. The C-terminal peptide is freely secreted, without any biological activity under physiological conditions. After binding of Shh on Ptch (Ptch1 or Ptch 2), the inhibitory effect of Ptch on Smo, another membranous protein, will be suppressed. The Smo activation activates Gli proteins that translocate thereafter into the nucleus and initiate target gene transcription. Several complex regulatory factors have been described in Shh pathway. Hedgehog interacting protein (HHIP) is a membranous protein that competes with Ptch for Shh binding [25]. Ptch receptor is also internalized in the cytosol after Shh binding, limiting in time the effects of its activation [26]. Suppressor of fused homolog (SUFU) protein binds to Gli proteins and retains them in the cytosol [27]. Gli3 can also be phosphorylated and induce inhibitor effect on transcription. The Zine finger of the cerebellum (ZIC) proteins interact with Gli proteins through their zinc-fingers domain, with alternative activating or inhibitor role [28,29].

Non-canonical activation of Shh pathway has also been described, especially in cancers [30]. Non-canonical activations of Shh pathway involve the activation of Gli proteins independently of Shh, Ptch, and Smo. Several bypass pathways are known as non-canonical activators of Shh pathways, such as the kinase pathway, through Ras-Raf-Mek activation. There is notably a close interaction between Shh and Epidermal Growth Factor receptor (EGFR) pathway [31]. EGFR pathway can induce Gli activation, through extracellular signal-regulated kinase (ERK) proteins [32,33]. TGF-β pathway can also activate Gli proteins without Ptch/Smo involvement [34], and other studies suggested Gli activation directly by protein kinase B-phosphoinositide 3-kinase (AKT-PI3K) [35] or tumor necrosis factor alpha (TNF-α) [36] pathways. The activation of non-canonical Hh pathway is thought to be responsible for Smo inhibitor resistant basal cell carcinomas [37].

The exact mechanism of the first two steps of canonical Shh signaling is surprisingly still enigmatic. How does Shh bind and activate Ptch and how does that further activate Smo? A co-receptor model seems to be in favor in terms of how Ptch is activated after Shh binding. The newly solved cryo-electron microscopy structures of human Ptch1 either alone or in complex with the N-terminal domain of Shh indicate that co-receptor binding to Ptch and ligand is physically plausible [38]. There are a couple of hypotheses regarding the action between activated Ptch and Smo [39]. Since Ptch has some sequence similarity with the bacterial resistance-nodulation-division (RND) permease, one theory is that Ptch transports a metabolite as an ion-driven small molecule pump to inhibit Smo. Another theory involves the regulation of lipid trafficking. Smo, also a transmembrane protein like Ptch, belongs to the classF/class6 superfamily of receptors. A classic example of this class of receptors is Frizzled, the receptor for Wingless-type (Wnt) ligands. Therefore, theoretically Smo could be regulated by a ligand. The prime suspect for this activating ligand has been some form of sterol. Initially, 20(S)-hydroxycholesterol was shown to be the strongest activating ligand for Smo [40,41]. Subsequently, cholesterol was thought to be a better candidate, which was found to co-crystalize with Smo [42,43]. Ptch also regulates Smo activation by transporting cholesterol [44]. Due to the critical position of Smo in the Shh pathway, it has been the principle target for inhibitor development. There are more than 20 inhibitors and 12 clinical trials (past and ongoing) targeting Smo [45]. Many inhibitors most likely bind Smo within the transmembrane domain [46].

It is now well accepted that the primary cilium is the primary location for Shh signal transduction [47]. Primary cilium is a thin, long protruding organelle that exists on most vertebrate cells. Ptch localizes at the membrane of primary cilium. At the inactive state, Smo is excluded from cilium, and SUFU sequesters Gli at the tip of the cilium. Once Shh binds to Ptch, Smo is allowed into the cilium, which represses SUFU, which in turn releases Gli. Gli then can be activated and translocates to the nucleus to activate downstream target genes. Studies have also shown that the movement of Shh pathway components is facilitated via intraflagellar transport (IFT) proteins. Hh pathway defects present common phenotypes as many ciliopathies (genetic disorders of the cellular cilia). Tumor cells with aberrant Hh signaling have also been reported to lack cilia [47,48,49,50,51,52,53].

Single, non-motile primary cilia are not to be confused with the motile cilia in some well-differentiated epithelial cells, especially those in the airway. Each of those cells can have hundreds of cilia on the apical cell surface. Jain et al. studied the relationship between primary and motile cilia in lung/airway epithelial cells [54]. They found primary cilia in cultured airway epithelial cell line (BEAS-2B), as well as in primary human tracheal epithelial cells obtained from donors for lung transplantation and mouse tracheal epithelial cells from C57BL/6J mice. Primary cilia are much more abundant once the tracheal cells became quiescent. Interestingly, proteins typically expressed in primary cilia can be found in motile cilia. It was also suggested that during lung development, motile ciliated cells originate from primary ciliated cells and primary cilia reoccurs on the basal side of adult epithelial cells during injury repair. Equally interestingly, not only secreted Shh ligand can be found right outside of the apical side (motile ciliated side) of the airway, Shh pathway components (Ptch, Smo, SUFU, Gli) were also found in motile cilia. Furthermore, Shh signaling in motile cilia was most likely via the non-canonical fashion involving cyclic adenosine monophosphate (cAMP) [55].

## 3. Shh Pathway Activation in Solid Tumors

Shh pathway is activated in many solid tumors. The first description of aberrant Shh pathway activation was in tumors with mutations in Shh pathway genes. The Gorlin syndrome is a rare hereditary disease, with an autosomal dominant transmission, characterized by the association of multiple basal cell carcinoma, medulloblastoma, and rhabdomyosarcoma. This disease is due to constitutive inhibitory Ptch mutations, inducing Smo activation [56,57]. Sporadic somatic mutations of SUFU were also described in medulloblastoma [58], and in basal cell carcinoma, there is often a mutation of Ptch or Smo [59].

Shh pathway can also be activated in solid tumors through a paracrine effect of Shh and canonical pathway activation. In small cell lung cancer (SCLC), the evaluation of the expression of Gli1 and Shh proteins shows high expression levels in SCLC cell lines and samples [60]. Inhibition of Smo induces inhibition of tumor growth in vivo [60,61]. A recent publication confirmed the importance of the Shh-dependent activation of the Shh pathway in SCLC [62]. Szczepny et al. showed that Shh is necessary and sufficient for the progression of SCLC in mice carrying both p53lox/lox and Rblox/lox alleles (producing SCLC in several months). Silencing Shh in these mice dramatically inhibits the tumor growth, whereas mice with induced overexpression of Shh develop larger tumors. Shh overexpression is associated with canonical activation of the Shh pathway and with highly proliferative SCLC tumors, with chromosomal instability and segmental aneuploidy [62]. In non-small cell lung cancer (NSCLC), Shh pathway is activated, and immunohistochemistry (IHC) studies have shown an homogenous over-expression of Shh pathway proteins in NSCLC samples, with a good correlation between each protein, suggesting a canonical activation of Shh pathway [63]. Other solid tumors have a non-canonical way of activation. In pancreatic carcinoma [64,65], breast carcinoma [66], bladder carcinoma [67], gastric carcinoma [68], esophageal carcinoma [36], melanoma [69], and glioblastoma [70], a Shh-independent activation of Gli1 or Gli2 has been occasionally described, mediated by Ras/Raf/Mek pathway, AKT/PI3K pathway, TGF-β or TNF-α pathways. Association of canonical and non-canonical activation of Shh pathway is also possible. In SCLC, even if the canonical activation seems fundamental as discussed before, an independent upregulation of cyclin B1 associated with Gli activation has been shown [62]. This combination of canonical and non-canonical activations in SCLC could explain the discordant results of clinical trials testing Smo-inhibitors in SCLC in association with chemotherapy [71,72]. In mesothelioma, the expression of different Shh-related proteins does not seem to be correlated [73], whereas mesothelioma cells were strongly dependent of Gli activation for survival and growth [74]. In this tumor type, protein kinase CK2 plays a key-role in the activation of Shh pathway [75]. However, the inhibition of Smo also induces a strong inhibition of tumor growth in vivo [73,76,77], suggesting a complex Shh activation profile in mesothelioma, mixing canonical and non-canonical activation.

## 4. Shh, Oncogenesis, and Cancer Stem Cells in Lung Cancer

In 1994, Lapidot et al. discovered a small population of cells in the peripheral blood of acute myeloid leukemia patients which has extremely strong tumorigenic ability [78]. This coined the concept of cancer stem cells, CSC. CSCs are believed to be the key for tumor initiation, proliferation, and recurrence, yet remain at a low proportion in tumors [79,80]. They are believed to be responsible for resistance to anti-tumor treatments, and in tumor spreading and metastatic process through EMT [79]. Several signaling pathways are known to be closely associated with CSCs. Among them, Hh, Wingless-type (Wnt), and Notch pathways are the main pathways activated in CSCs and responsible for their stock maintenance. The relation between Hh pathway and CSCs has been documented in many publications [81,82,83,84,85,86]. Just like for the discovery of CSCs, Hh pathway was found to be essential for the maintenance of CSCs in hematopoietic malignancies [87,88,89]. Dierks et al. and Zhao et al. both used a Bcr-Abl mouse model of CML (chronic myeloid leukemia) to demonstrate that Smo is required for the self-renewal ability of CSCs [87,88]. In their studies, Hh inhibitor cyclopamine inhibited human CML either in cell lines or primary patient samples. Similar findings have been observed in solid cancers, such as glioma (including glioblastoma), bladder, breast, colon, gastric, pancreatic, and prostate cancers [81,83,84,85,86,90,91,92]. Finally, Figeac et al. investigated stem cell features and expression of Hh pathway components in human lung fibroblasts isolated from histologically-normal parenchymal tissue from patients (non-smokers/non-Chronic obstructive pulmonary disease (COPD), smokers non-COPD, and smokers with COPD) who were undergoing surgery for lung tumor resection [93]. Lung fibroblasts expressed mesenchymal stem cells markers, differentiation ability, and immunosuppressive potential, but these properties were altered in lung fibroblasts from smokers and COPD patients. In these populations, Hh pathway was over-activated and responsible of the alteration of the stem cell phenotype of non-COPD and COPD smoker lung fibroblasts.

It is not surprising that there is a similar case in lung cancers. Watkins et al. [60] found that Shh and Gli co-expression in many small cell lung cancer (SCLS) samples as well as in SCLS cell lines. Cyclopamine again caused growth inhibition/arrest of SCLS cell line and was rescued by a constitutively mutated Gli1 construct. Cyclopamine was able to inhibit SCLS growth in a xenograft model. Vismodegib (GDC-0449), a Hh pathway inhibitor (Smo inhibitor), was shown to reduce cell growth for both SCLC and lung adenocarcinoma cell lines [58,82]. In NSCLC, Shh pathway plays a role at the very first steps of tobacco-induced oncogenesis, and inhibition of the Shh pathway is able to prevent the tobacco-induced tumor phenotype in cell lines [94]. Shh pathway is activated in CSCs by nicotine exposure [95]. Treatment of NSCLC cell lines by vismodegib completely inhibited the formation of tumor xenografts in nude mice [96]. Several works have also suggested a close interaction between Shh pathway and other CSC-related pathways, namely Wnt and Notch [97]. Conflicting results showed either activation of Wnt pathway by Shh pathway, or inhibitory effect of Shh pathway on Wnt, depending on cancer types [98,99,100,101,102]. However, it seems globally that Shh pathway acts upstream of Wnt activation in CSCs, at the very first steps of oncogenesis [103]. Recently, Giroux-Leprieur et al. have described the mechanisms of Shh activation in NSCLC [96]. They showed that CSCs produce a full-length, non-truncated Shh protein that exerts paracrine activity on other tumor cells. These CSCs, recognized by the full-length Shh protein on the cell membrane, represent often less than 0.5% of tumor cells in tumor samples, and are the signal source of Shh pathway activation in NSCLC. They have high oncogenic potential and are chemoresistant to cytotoxic chemotherapy. Inhibition of Shh pathway by vismodegib or by a Gli-inhibitor molecule induces a dramatic decrease of these CSCs’ population in vitro and in vivo [96]. A specific Shh-antibody, developed against the C-terminal peptide of the protein and targeting the full-length Shh protein, has shown promising anti-tumor activity in NSCLC in vivo [104].

## 5. Shh Pathway and Resistance to Chemotherapy

Chemotherapy remains the standard of care for many lung cancer patients, especially used as an adjuvant treatment in addition to surgery [105,106]. Unfortunately, some percentage of treated cancers will become resistant and recur. Standard chemotherapy agents for lung cancer include platinum drugs, taxane, etoposide, gemcitabine, vinorelbine, and pemetrexed. Many theories have been proposed to explain resistance and researchers are tirelessly seeking strategies to overcome it. Using a whole-genome synthetic lethal RNA interference screen, Marini et al. identified that the Activin pathway is essential for platinum resistance [107]. Blocking activin and growth differentiating factor 11 can significantly improve platinum induced cancer cell death.

Very recently, a group of researchers reported the use of nanotechnology in effort to overcome cancer cells’ drug resistance [108]. They devised a lipid membrane-coated silica carbon hybrid nanoparticle to target mitochondria. It specifically produces reactive oxygen species (ROS) in mitochondria under near-infrared (NIR) laser irradiation. This strategy was able to generate a therapeutic window and efficiently inhibit tumor growth without much toxicity. The drug-resistant mechanism, that is the target for the nanoparticle as well as many others, is a group of transporters-based efflux pumps, called ATP-binding cassette (ABC)-transporters [109]. In lung cancer, MRP family (also known as ABCC) or multiple drug resistance (MDR) P-glycoprotein family (also known as ABCB) transporters are most relevant [105]. CSCs are thought to a major contributor to resistance to cancer therapy and are known to express high levels of these ABC-transporters [110].

Shh pathway activation is closely associated with chemoresistance in bulk tumor cells as well as in CSCs. Inhibition of Shh pathway sensitizes in vitro cancer cells to chemotherapy, and Shh is associated with the expression of ATP-binding cassette (ABC)-transporters [111]. Moreover, Ptch1 was found to be able to act as a drug efflux pump in cancer cells, using the proton-gradient between intra- and extra-cellular spaces [112,113]. NSCLC cells that developed resistance to cisplatin, are sensitive to vismodegib, and have lower cisplatin IC50 with concomitant inhibition of Shh pathway [114,115]. Proportion of Shh+ CSCs is positively correlated in vitro with cisplatin IC50 [96]. A group in Canada combined gene expression modeling and siRNA screening in search of commodities that can enhance platinum sensitivity in lung cancer cells. Hh pathway was identified from the screen as one of the pathways [116]. Moreover, Shh pathway is associated with EMT, with a correlation between Gli, E-cadherin, and vimentin expressions in IHC [63,117]. NSCLC cells that acquired an EMT phenotype under TGF-β exposure are resistant to cisplatin, but inhibition of Shh pathway is able to sensitize the cells to cisplatin and decrease the expression of CSC phenotype [115]. A retrospective analysis of a NSCLC patients cohort (*n* = 36) treated with cisplatin-based chemotherapy in first line confirmed this correlation. Giroux-Leprieur et al. analyzed the expression of Gli1 and Gli2 by IHC, and showed that tumors with high-expression of Gli2 have more often early progression with chemotherapy, compared to other tumors [118]. Moreover, in vitro analyses (NSCLC cell lines and primary cultures of chemoresistant NSCLC samples) show that vismodegib treatment have synergic effect with cisplatin on cell survival in the most chemoresistant tumors cells [118]. 

## 6. Shh Pathway and Resistance to Tyrosine Kinase Inhibitors

Somatic mutations in the epidermal growth factor receptor (EGFR) is a driven oncogenic event found in 50% of Asian NSCLC patients and 10–15% of Caucasian patients [119,120]. EGFR pathway is related to non-canonical Shh activation, as discussed before, through the Ras-Raf-Mek cascade. Inhibition of Shh pathway in vitro has a synergistic effect with EGFR targeted therapies (tyrosine kinase inhibitors, TKIs) on NSCLC cell lines [121,122,123]. Somatic mutations of EGFR occur in around 15% of advanced NSCLC [124]. In 90%, mutations concern exon 19 or exon 21, related to high sensitivity to EGFR tyrosine kinase inhibitors (TKIs). However, all patients experienced progression (median 10–12 months). The main resistance mechanism is the acquisition of T790M mutation, but EMT has also been described as a major factor of secondary progression with EGFR TKI [125]. Shh pathway is closely associated with EMT [63,117]. Moreover, Shh pathway is associated with presence of EGFR mutations in lung adenocarcinoma [126]. Kim et al. performed IHC of Shh, Gli1, Gli2 and Gli3, and also ABCG2 in 166 early-stage lung adenocarcinomas. Shh expression was more frequent in lepidic adenocarcinoma and in the case of EGFR mutation. NSCLC cells with acquired resistance to EGFR TKIs have a high level of Smo expression, through Smo amplification [127]. Della-Corte et al. used in vivo xenograft models of EGFR mutated NSCLC treated with third-generation EGFR TKI (osimertinib) in association with a Mek inhibitor (selumetinib) [128]. They showed that Shh pathway is involved in resistance to this combo treatment, and that inhibition of Shh pathway inhibits proliferation, cell migration, and invasive properties of ex vivo resistant cultured cells. At last, in EGFR mutated NSCLC, Shh pathway seems to be also associated with MNNG HOS Transforming (MET) amplification, another well-known resistance mechanism to EGFR TKI [127,129].

Anaplastic lymphoma kinase (ALK) or ROS1 rearrangements (often translocation) are rare features in NSCLC (4% for ALK, 1% for ROS1 rearrangement) [124]. Specific TKIs have high efficiency in these situations, but progression occurs after 12–15 months of treatment. No published data have reported the role of Shh pathway in NSCLC with ALK or ROS1 rearrangement or in resistance to ALK/ROS1 TKIs. Shh pathway is activated in other tumors with ALK expression, as in large cell lymphoma [130,131]. Besides, ALK-EML4 translocation in NSCLC is associated to EMT and induces a CSC phenotype [132]. Several studies have also shown that EMT was associated with resistance to crizotinib (a first generation TKI targeting ALK and ROS1), through tumor hypoxia [133], activation of another tyrosine kinase receptor (AXL) [134,135,136] or TGF-β pathway [134]. In a cell line with ROS1 translocation, resistance to crizotinib was mediated by EMT and Twist1 activation [137]. Therefore, Shh pathway, related to EMT, seems to be a putative candidate to target to overcome resistance to ALK/ROS1 TKIs, similarly to EGFR TKIs.

## 7. Shh Pathway and Resistance to Radiotherapy

Radiation therapy is also a part of standard care for lung cancer. Locally advanced stage III NSCLC will receive thoracic radiation therapy together with chemotherapy whereas early stage or oligometastatic lesion could be treated with stereotactic body radiation therapy [106]. It is also commonly used for SCLC. Resistance to radiotherapy is a common phenomenon but the mechanism is not understood. Many in vitro studies have shown an implication of Shh pathway in resistance to radiotherapy. Cancer cells treated by radiotherapy present an activation of Shh pathway. A radioresistant osteosarcoma cell line clearly showed upregulation of Hh pathway and inhibition of Shh impaired its proliferation and invasive ability [138]. In glioma, RNAi against Histone deacetylase (HDAC) or a natural compound curcumin both seem to induce radiosensitivity whose actions depended on Shh pathway [139,140]. Pretreating hepatocellular carcinoma cell lines with Shh peptide rendered these cell lines more resistant to ionizing radiation [141]. Culture media from irradiated cells when added to naive tumor cells was able to protect them from the effects of radiation [141]. Shh protein was found in this media, and neutralization antibody reversed the protective effect of the media which suggests autocrine secretion of Shh responding to radiotherapy [141]. In cancer cell lines HT29 (colon cancer origin) and Panc1 (pancreatic origin), X-ray irradiation not only increased the expression of Shh and Gli1 at protein level, also induced the transcriptional activity of Gli1 [142]. This study employed a unique assay where the growth of un-irradiated cells was measured after being seeded on top of irradiated cells. The irradiated feeder cells stimulated the growth of the unirradiated cells at the X-ray dose that induced Shh/Gli. Inhibition of Shh pathway in the feeder cells reduced the growth of the unirradiated cells. This may suggest that Shh acts in a paracrine fashion in response to radiotherapy [95,96]. Similar in vitro results were observed in head and neck squamous carcinoma cells [138,143]. These authors also generated orthotopic xenograft tumors in nude mice using the head and neck carcinoma cells. Shh pathway inhibitor cyclopamine had a modest effect when combined with radiation. The authors also investigated tumor “repopulation” after radiation. Interestingly, radiation treated tumor stroma had the strongest ability to promote growth of fresh xenograft implants whereas tumor stroma treated with combined radiation and cyclopamine was much weaker, reminiscent of the paracrine effect seen in Reference [142]. In vivo inhibition of Shh pathway by Gli antagonist (GANT61), a Smo-inhibitor, has synergistic effect with radiotherapy in xenograft model of prostate cancer [144]. A monoclonal anti-Shh antibody, 5E1 or LED225 (Novartis) was able to increase the efficacy of radiation treatment in patient-derived xenograft models of cervical and esophageal cancer [145,146]. Clinically, expression of Shh pathway (Gli1 staining) was associated with the efficacy of esophageal patients treated with chemoradiation and surgery, in two cohorts of patients (*n* = 60 and *n* = 167) [147]. Moreover, activation of Shh pathway is associated with tumor relapse after radiotherapy in patients with head and neck carcinoma and cervical carcinoma [148,149]. In NSCLC, radiotherapy induced in vitro EMT and CSC phenotype [150], whereas Shh inhibition sensitized NSCLC tumors to radiation therapy, both in vitro and in vivo [151]. Prospective clinical trials are needed to evaluate the interest of targeting Shh pathway during radiotherapy.

## 8. Shh Pathway and Resistance to Immune Checkpoint Inhibitors

Recently, anti-cancer immunotherapy has taken a large place in the treatment strategy in NSCLC [152]. Immune checkpoint inhibitors (ICIs), anti-PD1 or anti-PD-L1 antibodies, have shown their efficacy, alone or in combination with chemotherapy, in several phase III randomized trials [153,154]. However, resistance mechanisms are still poorly understood with these treatments. Pham et al. have studied the efficacy of ICIs in two mice models of medulloblastoma (one Shh-dependent and one Shh-independent). They showed that ICIs had poor efficacy in the Shh-dependent model of medulloblastoma, whereas they kept good anti-tumor efficacy in the Shh-independent model [155]. Moreover, Wnt pathway, often associated with Shh pathway as discussed earlier [97], is associated with low immune infiltration and poor response to ICIs in cancer patients [156,157,158]. Using immunotherapy to target cancer stem cells has been an idea in the field for some time. High expression of PD-L1 on stem like cell population has been reported for head and neck squamous cell carcinoma (CD44^+^) and colon cancer (CD133^+^) [159,160]. When added to a gastric cancer derived cell line that possesses many stem cell characteristics, PD-1 ligand stimulated the expression of PD-L1 and enhanced the tumorigenicity of these cells [161]. Finally, a statistical study suggested that a sizable portion of solid tumors will benefit from combined therapy of oncogene targeting and immune checkpoint blockers. This includes Smo mutated/hedgehog pathway altered tumors [109].

## 9. Conclusions

**Figure 2:**
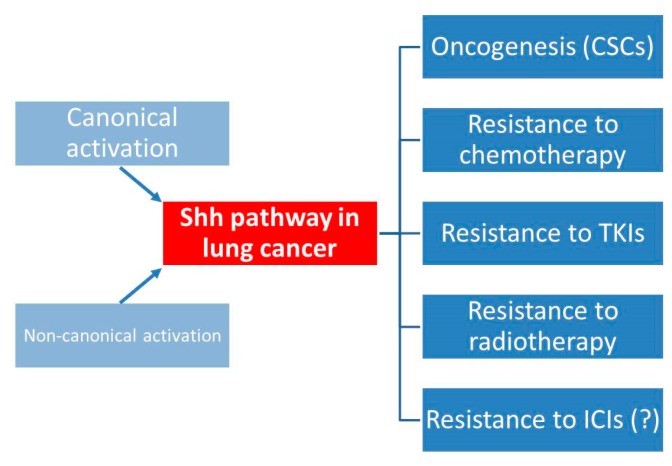
Shh activation in lung cancer and clinical implications. CSCs: cancer stem cells; TKIs: tyrosine kinase inhibitors; ICIs: immune checkpoint inhibitors. (?): not enough supporting evidence.

Shh pathway is involved in many ways in lung cancer (Figure 2). At the early step of oncogenesis, CSCs are the source of Shh pathway activation with a paracrine action on other tumor cells, leading to tumor growth and spreading through EMT. It is involved in resistance to all the main treatments in lung cancer: cytotoxic chemotherapy, radiotherapy, EGFR TKIs, and possibly to ICIs. Even if the canonical activation of Shh pathway seems predominant in lung cancer (NSCLC and SCLC), the possibility of non-canonical activation makes more complex the possible treatment strategies for Shh targeting. Further clinical trials in lung cancer should take into account this issue, and use not only Smo-inhibitors (already available in clinic) or Shh antibodies, but also Gli-inhibitors that are in development [76], to correctly cover all the ways of Gli activation. This would be the condition to reach sufficient efficacy of Shh targeting, and so increase the efficacy of current treatments in lung cancer patients.
